# The Distribution of Asian American Scholarship Awards Among Chinese, Indian, and Filipino Individuals

**DOI:** 10.3390/bs16060981

**Published:** 2026-06-12

**Authors:** A. Chyei Vinluan, Keith B. Maddox, Jessica D. Remedios

**Affiliations:** 1 Department of Psychology, Northwestern University, Evanston, IL 60208, USA; 2 Department of Psychology, Tufts University, Medford, MA 02155, USA; keith.maddox@tufts.edu (K.B.M.); jessica.remedios@tufts.edu (J.D.R.)

**Keywords:** Asian Americans, group typicality, scholarship, resource distribution

## Abstract

Perceptions of group typicality can shape how resources, such as scholarships, are distributed among group members. Across two studies, we predicted that East Asian applicants (e.g., Chinese) would be perceived as more typical of Asian Americans and thus more likely to be seen as worthy of an Asian American scholarship than their South Asian (e.g., Indian) and Southeast Asian (e.g., Filipino) counterparts. In Study 1, Chinese applicants were rated as more worthy of an Asian American scholarship than both Indian and Filipino applicants. Moreover, perceptions of Asian typicality predicted greater scholarship worthiness. In Study 2, we added a general scholarship condition that did not specify ethnicity to test whether Asian typicality influenced scholarship worthiness when the award was not designated for Asian Americans. However, we did not find the expected interaction between applicant ethnicity and scholarship type. Instead, we found only that the Chinese applicant was rated as more worthy of scholarships, regardless of scholarship type. Overall, perceptions of Asian typicality may guide judgements of scholarship worthiness in contexts where Asian identity is relevant.

## 1. Introduction

In the U.S., the term “Asian American” refers to a collection of individuals who represent a wide range of nationalities, ethnicities, and cultural backgrounds. The U.S. Census (U.S. Office of Management and Budget, 1997) defines ‘Asian’ as “a person with origins in any of the original peoples of East Asia, Southeast Asia, or South Asia, including countries like China, India, Japan, Korea, the Philippines, and Vietnam.” Asian Americans have the option to select one of these groups or write in their ethnic identity as they complete the U.S. Census. In 2023, 24.8 million individuals identified as Asian in the U.S. However, institutions within the U.S., such as governmental and educational institutions, often ignore this diversity by aggregating data from individuals of diverse Asian American ethnicities. For example, the National Science Foundation’s (2023) report on Diversity and STEM found that 10% of the STEM workforce is Asian American. However, statistics from the first college admissions cycle after the Supreme Court ended affirmative action show that Asian American college enrollment decreased by up to 6% at Yale University and Princeton University (Yam & Chaidez, 2024). In these examples, data are provided at the Asian American level—no further data are presented to describe the Asian ethnic groups represented within these trends (e.g., Chinese, Indian, Filipino).

The tendency for institutions to collapse data representing diverse Asian American ethnic groups mirrors how U.S. participants’ social perceptions of Asian Americans tend to ignore ethnic diversity. Research suggests that East Asian Americans (e.g., Chinese, Japanese, Korean) are perceived as more “typical” Asian Americans compared to South (e.g., Indian) and Southeast Asian Americans (e.g., Filipino, Vietnamese) (Goh & McCue, 2021; Lee & Ramakrishnan, 2020). Moreover, people tend to focus on more typical group members and overlook less typical group members (Cheryan & Markus, 2020; Smith & Zárate, 1992). In the present research, we conceptualize Asian identity as a racial identity and national origin identities (e.g., Chinese, Indian, Filipino) as ethnic identities. Race implies power, as in certain racial groups in the U.S. (i.e., White Americans) have power over other racial groups (i.e., Asian, Black, and Latino Americans) (Markus, 2008). In contrast, ethnicity implies a shared culture, history, and language associated with the national origin country (Markus, 2008). In our studies, we examine perceptions of Chinese, Indian, and Filipino individuals, corresponding to groups within East, South, and Southeast Asian Americans, respectively. These ethnic groups represent the three largest Asian ethnic populations within the U.S. (Jin, 2021; Krogstad & Im, 2025; U.S. Census Bureau, 2012). We recognize, however, that many Asian ethnic subgroups are left out of this kind of approach, indicating the need for further research to examine diversity among Asian Americans (Vinluan & Remedios, 2024).

Previous research has identified a range of consequences of focusing on typical group members while ignoring members perceived to be less typical of the group; for example, this bias impacts discrimination attributions (Goh et al., 2022; Pejic & Deska, 2025) and psychological sampling practices (Vinluan & Remedios, 2024). In this paper, we examined the consequences of focusing on group members who are perceived as typical in the context of resource distribution, specifically, *diversity awards* (Germano et al., 2021). Diversity awards are typically established to reduce economic inequality between racial minority groups, particularly Black, Latino, Indigenous, and Asian Americans, and White Americans (Germano et al., 2021). A common example is a scholarship for which only racial minority individuals are eligible, and that can reduce or eliminate costs associated with attending college or university. Indeed, Black and Latino American adults cite not being able to afford a four-year degree as a main reason that they did not attend college (Mora, 2022); thus, scholarships help to reduce such financial obstacles. Unfortunately, the distribution of traditional scholarships is biased toward majority-group members (Ginther et al., 2011, 2016; Hechtman et al., 2018; Lincoln et al., 2012). To combat this bias, institutions may designate funding specifically for racial minority groups. However, perceptions of group typicality often influence how resources are distributed to individuals *within* a racial group (Sanchez & Bonam, 2009; Good et al., 2013; Sanchez et al., 2011; Sanchez & Chavez, 2010; Wilton et al., 2013). Indeed, we expected East Asian American individuals (e.g., Chinese Americans) to be perceived as more worthy than South (e.g., Indian) and Southeast (e.g., Filipino) American individuals of an Asian American scholarship.

Moreover, if perceived group typicality shapes perceptions of the scholarship eligibility of racial minority group members, typicality should matter only when group membership is a criterion for applying for the scholarship. In other words, we reasoned that perceived Asian typicality should not matter when evaluating Asian American students for a general scholarship not designated specifically for Asian Americans.

### 1.1. Structural Influences on the Social Perceptions of Asian Americans

The U.S. government’s lack of acknowledgment of the ethnic diversity of Asian Americans and cultural portrayals of Asian Americans as predominantly East Asian parallel social cognitive biases in which East Asian Americans are perceived as typical of the Asian American group (Goh & McCue, 2021; Lee & Ramakrishnan, 2020). However, the practice of aggregating data across diverse Asian ethnic groups and presenting data at the Asian American level may hide inequalities within this group. For example, the statistic in which 24% of Asian Americans have an advanced degree compared to only 13% of all Americans (Shivaram, 2021) perpetuates the narrative that Asian Americans are academically successful and the *model minority* (Chun, 1995). However, analyzing the data by ethnic subgroup reveals that only 10% of Filipino Americans have an advanced degree, while 29% of Chinese Americans and 43% of Indian Americans have one (Shivaram, 2021). Further, presenting statistics in aggregate form may reduce awareness among people in the U.S. of inequalities within the Asian American category (Vinluan & Kraus, 2026). Taken together, this evidence suggests that people may overlook the ethnic diversity of the Asian American category, in part, because governmental and other institutions rarely collect and present data at the ethnic subgroup level. As one example of institutional actions recognizing the importance of how these data are presented, recently, the New York State legislature passed a bill requiring state agencies to collect information about Asian Americans at the ethnic subgroup level (New York State Assembly, 2021).

In addition to institutions overlooking the ethnic diversity of Asian Americans, cultural depictions of Asian Americans have primarily highlighted East Asian Americans. For example, an analysis of history textbooks in the U.S. showed that when children learn about Asian American history, they are more likely to learn about events in East Asian countries or prominent East Asian individuals than South or Southeast Asian countries or individuals (Suh et al., 2015). Additionally, when people watch an Asian American character in a television show or movie in the U.S., they are more likely to see a character of East Asian descent than a character of South or Southeast Asian descent (Yuen et al., 2021). Even the psychological field tends to highlight East Asian Americans. A literature review of psychological articles studying Asian identity revealed that 70% of Asian participants were of East Asian descent, in contrast to 18% of South Asian descent and 12% of Southeast Asian descent (Vinluan & Remedios, 2024).

Biases in institutional and cultural depictions of Asian Americans as primarily East Asian may contribute to social perceptions that reduce the Asian American category to typical or East Asian Americans. In the person perception literature, perceived typical group members are those who are considered by society as the category members who have the featural (i.e., facial characteristics or phenotype) and/or behavioral traits that are perceived to be stereotypical of that group (Devine & Baker, 1991; Hinzman & Maddox, 2017; Johnston & Hewstone, 1992; Johnston et al., 1994; Maddox, 2004; Maurer et al., 1995; Richards & Hewstone, 2001). Perceptions of typicality often have varying outcomes for group members. For example, when participants learn that an individual is the target of anti-Asian hate crimes, they rate East Asian individuals’ claims of discrimination as more credible and legitimate than when South Asian individuals claim they are targets of anti-Asian hate crimes (Pejic & Deska, 2025). Judgments of perceived Asian typicality mediated this effect: participants perceived East Asian individuals as more typical of Asian Americans than South Asian individuals, which led to ratings of East Asian individuals’ claims of discrimination as more legitimate than South Asian individuals’ discrimination claims. These findings suggest that outcomes for Asian Americans of varying ethnicities are shaped by whether they are perceived as typically Asian. In the current paper, we examined the role of perceived Asian typicality in the distribution of scholarships among Asian Americans.

### 1.2. The Role of Perceived Typicality in Scholarship Distribution

Diversity awards, like scholarships, are designed to alleviate the financial burden of attending college, but are more specifically aimed at reducing inequality in educational attainment between racial minority groups (e.g., Black, Latino, Indigenous, and Asian Americans) and White Americans (Germano et al., 2021). White and male students are more likely than racial minority and female students to be awarded non-diversity-centered scholarships, despite being equally qualified (Ginther et al., 2011, 2016; Hechtman et al., 2018; Lincoln et al., 2012). To offset these disparities, universities or institutions create scholarships that are designated for specific racial minority groups. For example, McDonald’s HACER National Scholarship program awards up to $100,000 to Hispanic/Latino students (McDonald’s Corporation, n.d.) to help increase the number of Hispanic/Latino Americans who have a bachelor’s degree in the U.S.

However, despite the goal of diversity awards to reduce inequality, disparities exist in their very distribution. Group members who are perceived as typical of the group are rated as more deserving of a diversity scholarship than atypical group members (Sanchez & Bonam, 2009; Good et al., 2013; Sanchez et al., 2011; Sanchez & Chavez, 2010; Wilton et al., 2013). For example, participants who rated a Latino applicant as typically Latino (i.e., because the applicant spoke Spanish) rated them as more deserving of affirmative action, more deserving of an internship for minority individuals, and more deserving of a scholarship for minority individuals as compared to an individual whom participants rated as less typically Latino (Sanchez & Chavez, 2010; Wilton et al., 2013). In another study, a Black monoracial applicant was perceived as more deserving of a racial minority scholarship than a Black/White multiracial applicant, who may be considered less typical of the Black category due to having multiple racial identities (Good et al., 2013; Sanchez et al., 2011; Sanchez & Bonam, 2009). These results suggest that the perceived typicality of a racial minority individual within their minority group guides perceivers’ beliefs about how minority resources should be distributed.

There has yet to be research on perceived typicality and scholarship distribution in the context of Asian Americans. Overall, similar to previous research, we hypothesized that participants would rate East Asian applicants as more deserving than South or Southeast Asian applicants of a scholarship designated for Asian American students. We further expected perceived Asian typicality to mediate this effect (e.g., Pejic & Deska, 2025). We additionally examined explanations for why perceived Asian typicality leads to perceptions of scholarship worthiness. Perceived typicality relates to perceptions of stereotypical ability (Maddox, 2004; Maddox & Gray, 2002) and group identification (Wilkins et al., 2010; Kaiser & Wilkins, 2010), both of which may be relevant when evaluating whether an applicant is deserving of a scholarship designated for Asian Americans. Group members who are perceived as typical are more likely than those perceived as atypical to be stereotyped and perceived in ways that are consistent with the overall group stereotype (Maddox, 2004; Maddox & Gray, 2002). Relevant to the present study, scholarships are often awarded based on academic merit, and Asian Americans are often stereotyped as intelligent (Fiske et al., 2002). Additionally, whether a racial minority applicant is perceived as deserving of a scholarship is based, in part, on how much they are perceived as identifying with their racial minority group (Good et al., 2010). Participants tend to assume that perceived typical group members identify more strongly with their group than perceived atypical group members (Wilkins et al., 2010; Kaiser & Wilkins, 2010). Therefore, we expected an East Asian scholarship applicant to be stereotyped as more intelligent and to be perceived as higher in group identification than a South or Southeast Asian applicant. As a result, an East Asian scholarship applicant may be perceived as more deserving of an Asian American scholarship.

Furthermore, judgments of group typicality are context-dependent (e.g., Roth & Shoben, 1983), meaning that perceptions of group typicality should only matter when the group is relevant to the evaluative domain. In other words, judgments of Asian typicality should only predict whether an Asian American candidate is awarded a scholarship if the scholarship is designated for Asian Americans. Activating “Asian American” as a category should also activate representations of typical Asian Americans or East Asian individuals (Goh & McCue, 2021; Lee & Ramakrishnan, 2020). However, if a scholarship does not activate a social category and, instead, indicates that anyone can apply, judgments of Asian typicality should not predict whether an Asian American candidate is awarded the more general scholarship (Ginther et al., 2011, 2016; Hechtman et al., 2018; Lincoln et al., 2012). We test this possibility in Study 2 by asking participants to rate how deserving Asian applicants are for either a scholarship specifically for Asian American students or a scholarship for all undergraduate students.

### 1.3. The Present Research

Across two studies, we tested the hypothesis that an East Asian (Chinese) applicant would be rated as more deserving of an Asian American scholarship than either a South (Indian) or Southeast (Filipino) Asian applicant. In Study 1, participants evaluated how deserving Chinese, Indian, and Filipino applicants were of an undergraduate scholarship meant for Asian American students. The applicants were portrayed as equally qualified for the scholarship but varied in their ethnicity. Furthermore, we examined three potential mediators: perceived Asian typicality, perceived intelligence, and perceived Asian identification. In Study 2, we examined whether the effects of perceived group typicality on award evaluations were limited to Asian American awards and predicted that the effects of typicality would not extend to a general or unrestricted award.

In our materials, the Chinese, Indian, and Filipino groups were chosen to represent East, South, and Southeast Asian Americans, respectively, given that these ethnicities are the three largest Asian ethnic populations within the U.S. (Jin, 2021; Krogstad & Im, 2025; U.S. Census Bureau, 2012). However, we acknowledge that selecting only three ethnicities limits the generalizability of our results and does not fully represent the ethnic diversity of the Asian American category.

Materials for both studies, including the data and analysis syntax, are available on the Open Science Framework: https://osf.io/k4xng/overview?view_only=b8a4adcf307c45bea581166b49d802d3.

## 2. Study 1

Participants were presented with the personal essays of Chinese, Indian, and Filipino applicants for an Asian American undergraduate scholarship and were asked to rate each essay on a series of Asian American stereotypes, perceived Asian typicality, perceived identification with the Asian American category, and deservingness of an Asian American scholarship. Overall, we hypothesized that the Chinese applicant would be rated as more deserving of the Asian American scholarship than the Indian and Filipino applicants (pre-registration: https://osf.io/nwzht/overview?view_only=17b08b410efb494facf20003c86067c8). Additionally, we tested the possibility that perceived Asian typicality will mediate this effect.

### 2.1. Method

#### 2.1.1. Participants and Design

The study design was within-subjects (*applicant ethnicity*: Chinese, Indian, Filipino). We conducted a power analysis using PANGEA (Power ANalysis for GEneral Anova designs; Westfall, 2015) to determine the sample size needed to achieve 80% power to detect a small-sized (*d* = 0.20) significant main effect of applicant ethnicity for a repeated-measures design with three conditions. The power analysis recommended that we recruit at least *N* = 525 participants. *N* = 623 participants completed the study on Amazon’s Mechanical Turk in exchange for $0.33^1^ USD. However, we specified in the pre-registration that we would only analyze data from participants who passed an attention check^2^. Additionally, to screen for potential bots, we excluded responses whose average timing and number of clicks were two or more standard deviations below the mean time spent and number of clicks (Buchanan & Scofield, 2018). This left us with a final sample of *N* = 545 participants (64.2% White, 9% Black, 7.5% Asian, 3.7% Latino; 46% men, 54% women; *M_age_* = 33.67, *SD_age_* = 10.80).

#### 2.1.2. Procedure

After consenting to participate in the study, participants were initially told they would evaluate a series of essays written by U.S. students applying to college. The essays had the same prompt: “Some students have a background, identity, interest, or talent that perhaps hasn’t always been important but, over time, has become so meaningful they believe their application would be incomplete without discussing it. If this sounds like you, then please share your story”. Participants were presented with three essays, in random order, written by Chinese, Indian, and Filipino applicants, respectively. Participants were asked to rate each applicant on a series of measures, including scholarship worthiness, before moving on to the next applicant. To clarify, participants were not told they were evaluating the applicants for a scholarship until after reading the essays, when they received items assessing scholarship-worthiness. After participants saw all three applicants’ essays, they completed a demographic survey and were debriefed.

#### 2.1.3. Essays

We created three different personal statement essays for this study to maintain our cover story that the Chinese, Indian, and Filipino applicants were different people, whose applications were randomly selected from a larger pool for the purposes of this study. To ensure that participants saw each essay and applicant race only once, there were three clustering conditions to counterbalance the essays. The content of each essay remained the same across clusters; for example, the content of essay 1 was the same for all participants. However, participants in clustering condition 1 saw that essay 1 was written by the Chinese applicant, essay 2 was written by the Indian applicant, and essay 3 was written by the Filipino applicant. Participants in clustering condition 2 saw essay 1 by the Filipino applicant, essay 2 by the Chinese applicant, and essay 3 by the Indian applicant. Finally, participants in clustering condition 3 saw essay 1 by the Indian applicant, essay 2 by the Filipino applicant, and essay 3 by the Chinese applicant. The author’s ethnic identities were revealed by references to either their Chinese, Indian, or Filipino identities throughout the essay. Pilot study results (see Supplementary Materials) revealed that there were some significant effects of applicant ethnicity on participants’ evaluations of the essays and/or the applicant—for the results, please see the Supplementary Materials. However, because the patterns were not consistent in terms of which applicant was rated as higher on perceived quality (e.g., clear and concise, well organized, minor grammatical errors) and content (e.g., scholarship, leadership, and community service), we decided to move forward with the pilot-tested essays as the applicant ethnicity manipulation.

#### 2.1.4. Measures

**Perceived Competence.** We measured perceived competence using items identified by Fiske et al. (2002) as indicative of competence: *competent, confident, capable, efficient*, *intelligent*, and *skillful*. These items were averaged to create a composite competence score (α_Chinese_ = .88, α_Indian_ = .90, α_Filipino_ = .89)^3^.

**Perceived Asian Identity.** We measured Asian identity using the following item, “The applicant strongly identifies with their Asian identity” on a 7-point scale (1 = *Strongly Disagree*, 7 = *Strongly Agree*).

**Perceived Asian Typicality.** We measured Asian typicality using the following item: “While there was no photograph of the applicant provided, we would like you to rate how Asian *you imagine* the applicant looks on a scale from ‘Less Typically Asian Looking’ [1] to ‘Very Typically Asian Looking’ [7]”.

**Asian American Scholarship Worthiness.** Participants were told that the applicants were applying for a university-sponsored Asian American scholarship. Specifically, participants read the following scholarship description: “This applicant is also submitting their essay to apply for the scholarship below. The University Asian American scholarship is a $2500 scholarship awarded to Asian undergraduate college students with demonstrated scholarship, leadership, and/or community service”. Participants were asked to rate the following, “This applicant should be awarded this scholarship,” “This applicant deserves this scholarship,” and “This applicant would be my first choice for this scholarship” (adapted from Sanchez & Bonam, 2009) on a 7-point scale (1 = *Strongly Disagree*, 7 = *Strongly Agree*). The items were averaged to create a composite Asian scholarship worthiness score (α_Chinese_ = .88, α_Indian_ = .91, α_Filipino_ = .88).

### 2.2. Results

The descriptive statistics collapsed across conditions for each measure, and the correlations between each measure are in Table 1.

#### 2.2.1. Pre-Registered Analysis Plan

We conducted repeated-measures ANOVAs to test for a significant main effect of applicant ethnicity on our dependent variables^4^. If the main effect was significant, we conducted planned simple comparisons with the Chinese applicant as the reference group. Bonferroni adjustments were then applied to post hoc comparisons to determine whether there was a significant difference in ratings of the Indian and Filipino applicants. Unless otherwise noted, results are consistent with our pre-registered hypotheses.

#### 2.2.2. Asian American Scholarship Worthiness

There was a significant main effect of applicant ethnicity on Asian scholarship worthiness, *F*(2, 1030) = 13.51, *p* < .0001, *η_p_*^2^ = .026. The Chinese applicant (*M* = 5.19, *SD* = 1.25) was rated as significantly more deserving of the Asian scholarship than both the Indian (*M* = 4.89, *SD* = 1.43), *F*(1, 515) = 24.56 *p* < .0001, *η_p_*^2^ = 0.046, and Filipino applicants (*M* = 4.99, *SD* = 1.30), *F*(1, 515) = 111.51, *p* = .0007, *η_p_*^2^ = .022. There was not a significant difference between ratings of the Indian and Filipino applicants, *p* = .224, 95% confidence interval (CI) [−0.04, 0.24].

#### 2.2.3. Perceived Asian Typicality

There was a significant main effect of applicant ethnicity on Asian typicality *F*(2, 1022) = 71.00, *p* < .0001, *η_p_*^2^ = .122. The Chinese applicant (*M* = 5.52, *SD* = 1.22) was rated as significantly more typically Asian than both the Indian (*M* = 4.60, *SD* = 1.75), *F*(1,511) = 112.85, *p* < .0001, *η_p_*^2^ = .181, and Filipino applicants (*M* = 4.96, *SD* = 1.40), *F*(1,511) = 71.59, *p* < .0001, *η_p_*^2^ = .123. The Filipino applicant was rated as more typically Asian than the Indian applicant, *p* < .0001, 95% CI [0.17, 0.55].

#### 2.2.4. Perceived Competence

Inconsistent with our pre-registered hypotheses, there was not a significant main effect of applicant ethnicity on perceived competence, *F*(2, 1030) = 1.64, *p* = .195, *η_p_*^2^ = .003. Participants’ ratings of the applicant as competent did not significantly differ by ethnicity (Chinese: *M* = 5.47, *SD* = 0.89; Indian: *M* = 5.47, *SD* = 0.91; Filipino: *M* = 5.42, *SD* = 0.94).

#### 2.2.5. Perceived Asian Identity

There was a significant main effect of applicant ethnicity on Asian identity, *F*(2, 984) = 27.16, *p* < .0001, *η_p_*^2^ = .052. The Chinese applicant (*M* = 5.37, *SD* = 1.30) was rated as having a significantly stronger Asian identity than both the Indian (*M* = 4.80, *SD* = 1.67), *F*(1, 492) = 49.40, *p* < .0001, *η_p_*^2^ = .091, and the Filipino applicants (*M* = 5.13, *SD* = 1.41), *F*(1, 492) = 11.25, *p* = .0009, *η_p_*^2^ = .091. The Filipino applicant was rated as identifying significantly more strongly with their Asian identity than the Indian applicant, *p* = .0001, 95% CI [0.14, 0.51].

#### 2.2.6. Exploratory Parallel Mediation Analysis

We did not pre-register a plan to conduct a mediation analysis. However, we decided to conduct an exploratory parallel mediation analysis to test whether both perceived Asian identity and typicality mediated the relationship between applicant ethnicity and Asian scholarship worthiness. Specifically, we conducted a cross-classified multilevel mediation analysis using Bayes estimation on Mplus (Luo, 2017; Muthén & Muthén, 1998–2017) with applicant ethnicity dummy coded as the predictor variable (the reference condition was the Chinese applicant), perceived Asian typicality as the first mediator variable, perceived Asian identity as the second mediator variable, and Asian American scholarship worthiness as the outcome variable. We did not include perceived competence in the analysis because the ANOVA results indicated that there was not a significant difference in perceived competence ratings by applicant ethnicity.

**Figure 1:**
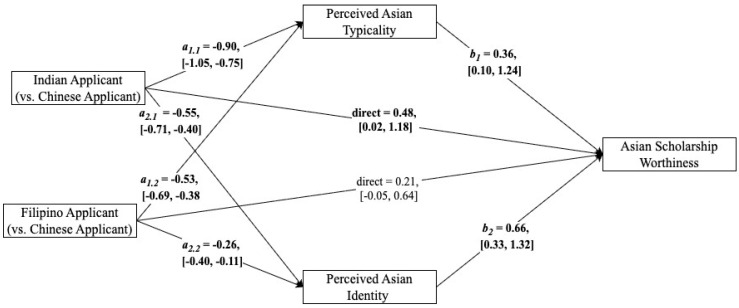
Study 1 parallel mediation results. Bolded text indicates significant effects, values in brackets represent 95% confidence interval.

Both ethnicity predictor variables were negatively associated with perceived Asian typicality and identity. See Figure 1. Specifically, ethnicity (Chinese vs. Indian) significantly predicted Asian typicality (β = −0.90, 95% CI [−1.05, −0.75]), Asian identity (β = −0.55, 95% CI [−0.71, −0.40]), and Asian scholarship worthiness (β = 0.48, 95% CI [0.02, 1.18]). Similarly, ethnicity (Chinese vs. Filipino) significantly predicted Asian typicality (β = −0.54, 95% CI [−0.69, −0.38]) and Asian identity (β = −0.26, 95% CI [−0.40, −0.11]) but not Asian scholarship worthiness (β = 0.21, 95% CI [−0.05, 0.64]). The indirect effects of ethnicity (Chinese vs. Indian) on scholarship worthiness via typicality (*indirect effect* = −0.33, 95% CI [−1.07, −0.09]) and identity (*indirect effect* = −0.36, 95% CI [−0.81, −0.16]) were significant, as the 95% confidence intervals did not include zero. This was also the case for the indirect effects of ethnicity (Chinese vs. Filipino) on scholarship worthiness through typicality (*indirect effect* = −0.20, 95% CI [−0.66, −0.05]) and identity (*indirect effect* = −0.16, 95% CI [−0.40, −0.05]). These findings suggest that both Asian typicality and Asian identity partially mediated the relationship between applicant ethnicity (Chinese vs. Indian) and scholarship worthiness and fully mediated the relationship between applicant (Chinese vs. Filipino) and scholarship worthiness.

### 2.3. Discussion

In Study 1, we found that a Chinese applicant for a diversity scholarship for Asian Americans was rated as more deserving of the scholarship than equally qualified Indian and Filipino applicants. Contrary to our expectations, we did not find that perceptions of intelligence significantly differed by applicant ethnicity. However, participants did rate the Chinese applicant as more typical of Asian Americans and more strongly identified with the Asian American group than both the Indian and Filipino applicants. An exploratory parallel mediation analysis revealed that participants’ ratings of the Chinese applicant as more typically Asian and identifying more with the Asian group related to participants’ perceptions of how worthy the Chinese applicant was of the scholarship in comparison to the Indian and Filipino applicants.

## 3. Study 2

In Study 2, we aimed to pinpoint the role of group typicality in judgments of diversity scholarship worthiness by experimentally manipulating a mechanism expected to make typicality perceptions salient. Specifically, we manipulated the type of scholarship to which the Asian applicants applied. The first scholarship type was the Asian American undergraduate scholarship described in Study 1. We expected that, in the context of this scholarship, group membership is activated and typicality judgments are relevant. We expected to replicate findings from Study 1 and discover that the Chinese applicant would be rated as more deserving of the Asian American scholarship than both the Indian and Filipino applicants. However, the second scholarship type was an unrestricted undergraduate scholarship. This scholarship was described as being for undergraduate students in general. In this context, lacking the activation of a social group or category, we did not expect group typicality to matter. As a result, we did not expect to observe significant differences in scholarship deservingness for a general undergraduate scholarship among the Asian applicants (pre-registration: https://osf.io/q5jg3/overview?view_only=ad2f253a79d1464eb4e5a7a05b72f8ae).

### 3.1. Method

#### 3.1.1. Participants and Design

The study was a 2 (*scholarship type:* Asian undergraduate, general undergraduate) × 3 (*applicant ethnicity:* Chinese, Indian, Filipino) mixed-model design with applicant ethnicity as a within-subjects factor. A power analysis using PANGEA (Westfall, 2015) determined that the sample size needed to achieve 80% power to detect a small-sized effect (*d* = 0.20) for a significant interaction between scholarship type and applicant ethnicity was at least *N* = 540 participants. Notably, the effect size from Study 1 was small (*d* = 0.30). We recruited *N* = 750 participants on Amazon’s Mechanical Turk in exchange for $0.33 USD^5^. However, we specified in the pre-registration that we would analyze data from only participants who passed a series of attention checks and screen out potential bots using the same criteria as in Study 1. This left us with a final sample of *N* = 561 participants (70.8% White, 10.6% Black, 6% Asian, 4.6% Latino; 41.9% men, 56.7% women; *M_age_* = 35.49, *SD_age_* = 12.46).

#### 3.1.2. Procedure

The procedure, essays, and items used in Study 2 were the same as in Study 1, with the exception that participants were randomly assigned to either the *Asian* or *general undergraduate scholarship* condition. Participants assigned to the *Asian undergraduate scholarship* condition were told that the applicants were applying for a “$2500 scholarship awarded to Asian undergraduate college students with demonstrated scholarship, leadership, and/or community service.” Participants assigned to the *general undergraduate scholarship* condition were told that the applicants were applying for a “$2500 scholarship awarded to undergraduate college students with demonstrated scholarship, leadership, and/or community service.”

### 3.2. Results

The descriptive statistics collapsed across conditions for each measure, and the correlations between each measure are in Table 2.

#### 3.2.1. Pre-Registered Analysis Plan

For scholarship worthiness, we conducted a 2 × 3 mixed-model ANOVA to test for a significant interaction between scholarship type and applicant ethnicity. If the interaction was significant, we planned to conduct simple comparisons within each scholarship condition with the Chinese applicant as a reference group. Bonferroni adjustments would then be applied to post hoc comparisons to determine whether there was a significant difference in ratings between the Indian and Filipino applicants within and between each scholarship type condition.

For the competence, identity, and typicality items, we were primarily interested in a significant main effect of applicant ethnicity to determine if we replicated the findings from Study 1. Each item was analyzed using a 2 × 3 mixed-model ANOVA; however, only the main effect of applicant ethnicity will be reported here, as we did not have a priori hypotheses regarding interactions (see Supplementary Materials for complete results). Unless otherwise noted, results are consistent with our pre-registered hypotheses.

#### 3.2.2. Scholarship Worthiness

There was not a significant main effect of scholarship type, *F*(1, 559) = 0.13, *p* = .720, *η_p_*^2^ = .0002. However, there was a significant main effect of applicant ethnicity on scholarship worthiness, *F*(2, 1118) = 3.66, *p* = .026, *η_p_*^2^ = .007. Overall, the Chinese applicant (*M* = 4.89, *SD* = 1.34) was rated as significantly more deserving of a scholarship (regardless of scholarship type) than both the Indian (*M* = 4.74, *SD* = 1.33), *F*(1, 559) = 6.98, *p* = .008, *η_p_*^2^ = .012, and Filipino applicants (*M* = 4.79, *SD* = 1.33), *F*(1, 559) = 3.06, *p* =.018, *η_p_*^2^ = .005.

We predicted a significant interaction between scholarship type and applicant ethnicity, wherein applicant ethnicity would significantly affect scholarship worthiness only in the Asian scholarship condition (in which typical Asian representations should be salient). However, there was no significant interaction between scholarship type and applicant ethnicity on scholarship worthiness, *F*(2, 1118) = 0.78, *p* = .460, *η_p_*^2^ = .001. Despite the lack of significant interaction, we deviated slightly from our planned analyses to compare applicant ethnicity ratings within scholarship type. This revised analysis plan enabled us to examine our pre-registered prediction that the Chinese applicant would seem most worthy only in the Asian scholarship condition. We examined the data using Bonferroni-adjusted post hoc comparisons^6^. Indeed, in the *Asian undergraduate* scholarship condition, the Chinese applicant (*M* = 4.95, *SD* = 1.36) was perceived as more deserving of the scholarship than the Indian applicant (*M* = 4.73, *SD* = 1.37), *p* = .028, 95% CI [0.02, 0.41], *d* = 0.152, but not the Filipino applicant (*M* = 4.79, *SD* = 1.32), *p* = 0.162, 95% CI [−0.04, 0.35], *d* = 0.121. There was no significant difference between the Indian and Filipino applicants, *p* = 1.00, 95% CI [−0.26, 0.14], *d* = −0.45.

However, in the *general undergraduate* scholarship condition, there were no significant differences in scholarship worthiness between the Chinese (*M* = 4.83, *SD* = 1.33) and Indian applicants (*M* = 4.74, *SD* = 1.28), *p* = .818, *d* = 0.072, 95% CI [−0.10, 0.28], nor between the Chinese and Filipino applicants (*M* = 4.79, *SD* = 1.33), *p* = 1.00, *d* = 0.029, 95% CI [−0.15, 0.23]. Additionally, there was no significant difference between the Indian and Filipino applicants, *p* = 1.00, *d* = −0.35, 95% CI [−0.24, 0.14]. Thus, despite the lack of a significant race × scholarship interaction effect, the pattern of the post hoc comparisons fits well with our pre-registered hypothesis.

##### Equivalence Testing

The pre-registration specified that we would conduct equivalence testing to determine if participants rated the Chinese, Indian, and Filipino applicants equally within the general undergraduate scholarship condition. As a reminder, we expected the applicants to seem similarly worthy for a general scholarship, assuming that the general scholarship does not activate typical representations of Asian identity. Using the two one-sided tests procedure to test for equivalence (Lakens et al., 2018) and setting our equivalence bound based on Cohen’s *d* = ±0.152^7^, the Chinese/Indian comparison was not significantly within the equivalent bounds, *t*(293) = 1.38, *p* = .085, suggesting that scholarship worthiness ratings between the Chinese and Indian applicants were not equivalent. However, the Chinese/Filipino comparison was significantly within the equivalent bounds, *t*(290) = 2.10, *p* = .018, suggesting that scholarship worthiness ratings between the Chinese and Filipino applicants were equivalent. Additionally, the Indian/Filipino comparison was significant within the equivalent bounds, *t*(290) = 2.02, *p* = .023, suggesting the scholarship worthiness ratings between the Indian and Filipino applicants were equivalent. Although somewhat mixed, the findings suggest that, for most comparisons, applicant ratings were equivalent within the general scholarship condition.

#### 3.2.3. Perceived Asian Typicality

There was a significant main effect of applicant ethnicity on Asian typicality, *F*(2, 1110) = 95.89, *p* < .0001, *η_p_*^2^ = .147. The Chinese applicant (*M* = 5.45, *SD* = 1.28) was rated as significantly more typical of the Asian group than both the Indian (*M* = 4.42, *SD* = 1.76), *F*(1, 555) = 156.70, *p* < .0001, *η_p_*^2^ = .220, and Filipino (*M* = 4.82, *SD* = 1.40) applicants, *F*(1, 555) = 87.70, *p* < .0001, *η_p_*^2^ = .136. The Filipino applicant was also rated as more typically Asian than the Indian applicant, *p* < .0001, 95% CI [0.22, 0.57].

#### 3.2.4. Perceived Competence

Inconsistent with our pre-registered hypotheses, we did not find a significant main effect of applicant ethnicity on perceived competence, *F*(2, 1118) = 0.72, *p* = .487, *η_p_*^2^ = .001, Participants’ ratings of the applicant as competent did not significantly differ by ethnicity (Chinese: *M* = 5.40, *SD* = 0.91; Indian: *M* = 5.41, *SD* = 0.88; Filipino: *M* = 5.37, *SD* = 0.90).

#### 3.2.5. Perceived Asian Identity

There was a significant main effect of applicant ethnicity on perceived Asian American identity, *F*(2, 1080) = 51.09 *p* < .0001, *η_p_*^2^ = .086. As in Study 1, the Chinese applicant (*M* = 5.20, *SD* = 1.39) was rated as having a significantly stronger Asian identity than both the Indian (*M* = 4.46, *SD* = 1.76), *F*(1, 540) = 85.35, *p* < .0001, *η_p_*^2^ = .136, and Filipino applicants (*M* = 5.06, *SD* = 1.43), *F*(1, 540) = 3.96, *p* = .047, *η_p_*^2^ = .007. The Filipino applicant was rated as identifying more strongly with their Asian identity than the Indian applicant, *p* < .0001, 95% CI [0.41, 0.81].

### 3.3. Discussion

Inconsistent with our hypothesis, we did not find a significant interaction between applicant ethnicity and scholarship type on scholarship worthiness. Instead, overall, the Chinese applicant was rated as more deserving or worthy of a scholarship than the Indian and Filipino applicants. However, we decided to test our pre-registered hypotheses (despite not finding a significant interaction) by conducting a series of post hoc comparisons and equivalence tests. The findings suggest that the Asian scholarship, more so than the general scholarship, likely activated prototypical representations of Asian identity. The Chinese applicant seemed to be perceived as most worthy of an Asian scholarship, whereas the applicants were perceived as similarly worthy of the general scholarship (one exception occurred in which we could not confirm the equivalence of ratings of the Chinese and Indian applicants for the general scholarship).

We additionally conducted parallel mediation analyses, revealing that neither Asian typicality nor identity significantly mediated the effect of ethnicity on overall scholarship worthiness and scholarship worthiness by scholarship type (see Supplementary Materials for detailed analyses). This result was inconsistent with Study 1. However, we did replicate findings from Study 1, that the Chinese applicant in Study 2 was perceived as more typically Asian and as identifying more strongly with their Asian identity than both the Indian and Filipino applicants.

## 4. General Discussion

The extent to which category members are perceived as typical or atypical of their category often has consequences for important, real-world outcomes, such as resource distribution. In the present research, we examined resource distribution in the context of diversity scholarships. The studies examined judgments of Asian American applicants who varied in ethnic identity and, theoretically, in how well they represented a prototypical member of the Asian American group. In Study 1, we found that a Chinese applicant was rated as more deserving of a scholarship meant for Asian American undergraduate students than an Indian and a Filipino applicant. Perceptions of how typical the applicant was of the Asian American category and how strongly the applicant identified with the Asian American category correlated with perceptions of scholarship worthiness. The Chinese applicant was rated as more typically Asian and identifying more strongly as Asian, which predicted the Chinese applicant as more deserving of the Asian American scholarship than the other applicants. Findings from Study 2 showed that the Chinese applicant was perceived as generally more deserving of a scholarship than the Indian and Filipino applicants, regardless of whether the award was designated for Asian Americans or was unrestricted and open to all undergraduate students.

However, we did not have evidence from Study 2 that Asian typicality plays a role in decisions when the scholarship is designated for only Asian Americans and not when the scholarship is unrestricted, given that we did not find the expected significant interaction between applicant ethnicity and scholarship type on scholarship worthiness. We expected that findings among participants in the Asian American scholarship condition in Study 2 would replicate findings from Study 1. However, we expected that in the unrestricted, general scholarship condition, the applicants’ perceived scholarship worthiness would be equivalent. Instead, we found that, across both scholarship conditions, the Chinese applicant was rated as more deserving of the Asian American scholarship than the Indian applicant. Additionally, the equivalence tests within the unrestricted, general scholarship condition were inconclusive. The Chinese–Filipino and Indian–Filipino comparisons were equivalent, while the Chinese–Indian comparison was not equivalent. Relatedly, in Study 2, we found that the Chinese applicant was rated as more deserving of the Asian American scholarship than only the Indian applicant, unlike in Study 1, where we found that the Chinese applicant was rated as more deserving than both the Indian and Filipino applicants. Therefore, we were also unable to completely replicate the parallel mediation findings from Study 1. Thus, we cannot conclude from Study 2’s findings that perceptions of Asian typicality of the scholarship candidate mattered in only the Asian American scholarship condition. Nevertheless, previous research suggests that judgments of group typicality are context-dependent (e.g., Roth & Shoben, 1983); thus, future research is needed to test if this is the case for judgments of the scholarship worthiness of Chinese, Indian, and Filipino American scholarship applicants. Additionally, our Study 2 findings unfortunately cannot disentangle whether the mechanism for Asian American scholarship worthiness is perceived Asian typicality, specifically, or perceived fit with the Asian American category. Though closely related, typicality refers to the degree to which a target group member is close to the standard group member, whereas fit refers to whether a target group member is consistent with the social group (e.g., Bless & Wänke, 2000; Craig & Bodenhausen, 2018). Therefore, we recommend that future research test these two mechanisms in the context of resource distribution among Asian Americans.

Our findings replicate previous research showing that individuals who are perceived as typical of the category are more likely to be awarded scholarships or resources designated for their category than atypical category members (Sanchez & Bonam, 2009; Good et al., 2013; Sanchez et al., 2011; Wilton et al., 2013). In both studies, the Chinese applicant was rated as more typical of the Asian American category than the Indian and Filipino applicants, in line with prior work suggesting that East Asian Americans are perceived as typical Asians while South and Southeast Asian Americans are perceived as atypical Asians (Goh & McCue, 2021; Lee & Ramakrishnan, 2020). On one hand, the results of Study 2 may suggest that a mechanism by which typical category members are perceived as most deserving of resources in general, even when those resources are disconnected from, or fail to activate, is group identity. Indeed, the Chinese applicant was rated as more deserving of a scholarship than Indian and Filipino applicants, regardless of the nature of the scholarship (identity-specific or general). However, it is unclear why this would be the case. Typicality is not necessarily related to merit.

That said, when judging who deserves a scholarship, participants may think of academic merit or the perceived intelligence of an applicant. Asian Americans are stereotyped as intelligent (Fiske et al., 2002) and as being academically successful (i.e., the model minority myth; Chun, 1995). Thus, regardless of the details of the scholarship, such as who is eligible, participants may activate perceived Asian typicality in an academic/award context. Specifically, to the extent that participants judged the Chinese applicant as typical of Asian Americans, the Chinese applicant may have also seemed intelligent and, thus, deserving of the scholarship. Despite this reasoning, we did not find evidence in either study that the Chinese applicant was rated as more intelligent than the Indian and Filipino applicants. In addition, we did not find significant differences by applicant ethnicity for other stereotypes specific to Asian Americans (see Supplementary Materials), such as perceived foreignness, which we would have expected to vary by Asian ethnic subgroup (e.g., Goh & McCue, 2021; Vang, 2025). This could be for several reasons, the first being that we provided participants with individuating information (e.g., a personal statement), making participants less likely to stereotype the applicants (Fiske & Neuberg, 1990). The other reason may be that we asked participants to stereotype the individual applicant rather than the overall ethnic subgroup (i.e., “the applicant is intelligent” versus “Chinese Americans are intelligent”). Participants may be more likely to stereotype groups rather than individuals (Cooley & Payne, 2019).

In addition to perceived Asian typicality, we found that perceived Asian identification was a potential mechanism for perceived Asian scholarship worthiness. The present research conceptually replicates findings that perceptions of group typicality are related to perceptions of group identification (Wilkins et al., 2010; Kaiser & Wilkins, 2010). Specifically, the Chinese applicant was rated as identifying more strongly with the Asian American category than the Indian and Filipino applicants. Participants appeared to assume that the more typical the applicant was of the Asian American category, the more strongly the applicant would identify with being Asian American, and as a result, the more deserving the applicant should be of the Asian American scholarship. Thus, it seems like participants use perceived racial minority identification to determine whether an applicant is worthy of a resource meant for racial minority groups. This also brings up an interesting question from the perspective of Asian Americans: do Asian Americans who are perceived as more typical of the category more strongly identify as being Asian? In other words, do East Asian Americans (e.g., Chinese, Japanese, Korean) identify more with being Asian American than South (e.g., Indian) or Southeast Asian Americans (e.g., Filipino, Vietnamese)? Research examining Asian Americans’ perceptions of Asian typicality suggest that South Asian Americans perceive their own ethnic subgroup as Asian in the same way that East Asian Americans perceive East Asian subgroups (Lee & Ramakrishnan, 2020). Whether or not this also means that South Asian Americans and East Asian Americans report similarly high levels of Asian identification, future research should examine the role of perceived and actual group identification in interracial interactions involving individuals aligned with various Asian American ethnicities.

Finally, our findings demonstrated (to some extent) that perceptions of group typicality were most relevant when the group was salient (Roth & Shoben, 1983). Specifically, in Study 2, perceived scholarship worthiness generally did not differ between the Chinese, Indian, and Filipino applicants when judgments related to a general, unrestricted scholarship. In this condition, our findings suggest that perceptions of Asian typicality were perhaps less relevant, given that the idea of fit with an ‘Asian American’ category was not activated among participants. One interpretation of these data may be to question the utility of diversity scholarships in the context of diverse and heterogeneous racial groups, like Asian Americans. In an effort to ameliorate racial disparities between White and Asian Americans, diversity scholarships may, ironically, exacerbate inequalities between East Asian Americans and members of less privileged Asian ethnic groups. However, we would caution against the conclusion that institutions should do away with diversity scholarships for this group. Indeed, this solution presents an issue: atypical Asian Americans are also less likely to be awarded general scholarships in comparison to White applicants (Ginther et al., 2011, 2016). Therefore, an alternative, more equitable solution may be to create scholarships or resources specifically for Indian and Filipino Americans. We acknowledge that there are expected challenges to creating such granular diversity scholarships, such as finding funding. But to overcome the bias that East Asian Americans may be judged as more worthy of diversity scholarships than South or Southeast Asian Americans, challenges to supporting more marginalized Asian American ethnic groups, particularly Southeast Asian Americans, must be confronted (Shivaram, 2021; Vinluan & Kraus, 2026).

### Limitations and Future Directions

Our studies have several limitations, offering potential avenues for future research. In both studies, we limited the Asian American ethnicities represented in our research to only three: Chinese, Indian, and Filipino. Our results allow us to make conclusions about how only Chinese, Indian, and Filipino individuals are perceived in the context of diversity scholarships. However, the Asian American group consists of more than 20 Asian ethnicities; therefore, it is unclear if our findings generalize to other East, South, and Southeast Asian ethnicities. This paper may provide a methodological framework for future research examining perceptions of diverse Asian ethnicities. Within our social cognitive theoretical lens that East Asians are perceived as typically Asian, we would expect applicants of East Asian ethnicities to be considered more deserving of an Asian American award than applicants of South and Southeast Asian ethnicities. However, it is possible that studies examining perceptions of different Asian ethnicities may discover different patterns. For example, while household income greatly varies by Asian American ethnic subgroup, the household incomes for Chinese, Indian, and Filipino Americans are still higher than the overall U.S. household median income (Krogstad & Im, 2025). Therefore, if we had selected ethnic subgroups whose household incomes are lower than the overall U.S. household median income (e.g., Mongolian, Burmese), candidates with more obvious funding needs may have been perceived as highly deserving of an Asian American scholarship. However, future research can test this possibility and more directly test the interaction between socioeconomic status and Asian ethnic subgroups on scholarship worthiness. Overall, we would expect different stereotypes and perceptions of Asian typicality to be activated depending on the Asian ethnicity of the applicant, impacting the extent to which the applicants are perceived as deserving of a scholarship. Therefore, it is possible that, for example, despite both Filipino and Vietnamese Americans being considered Southeast Asian ethnicities, there may be differences in how typically Asian American these two ethnicities are considered.

There are also ecological validity limitations for our study materials. First, the personal essay manipulations in both studies were purposely written to be similar in content to control for potential essay-related confounds. However, in reality, applicants will answer essay prompts differently, and some of those differences (e.g., related to lived experiences) may be confounded with Asian ethnicity, which will influence perceived scholarship worthiness. Second, participants were purposely given very limited information about the scholarship to which the applicants were applying. For example, we did not inform participants as to the purpose of the scholarship. Many academic scholarships targeted toward specific racial groups are meant to increase the representation and to alleviate disparities in higher-education-related outcomes between White Americans and the targeted groups. If we had informed participants of the purpose of the scholarship, we would expect different outcomes. Specifically, if participants were told that the scholarship was meant to increase the representation of students with identities that have been historically underrepresented in higher education, it is possible that we would not see significant differences between applicant ethnicity on scholarship worthiness, given narratives that Asian Americans, as a whole, are academically successful (e.g., model minority myth, Chun, 1995). Future research can test this hypothesis and, more broadly, how deserving Asian American applicants are of scholarships or awards meant for students from underrepresented backgrounds. This is especially important given that, in reality, not all Asian ethnic subgroups are well represented in higher education (Jin, 2021; Krogstad & Im, 2025; Shivaram, 2021), a fact which U.S. participants are generally unaware of (e.g., Vinluan & Kraus, 2026). If underrepresented Asian Americans (e.g., Southeast Asian Americans) are deemed as not worthy of scholarships meant for students from underrepresented backgrounds, then interventions may be needed to increase awareness of variation in Asian ethnic subgroup representation in higher education.

Another limitation is that the gender identity of the applicant was not specified in the essays. All the essays—the applicant ethnicity manipulation—were written from the perspective of the applicant. Based on gendered race theory (Johnson et al., 2012), stereotypes about Asian Americans and women are more consistent than stereotypes about Asian Americans and men. For example, both Asian Americans and women are stereotyped as *feminine* (Ghavami & Peplau, 2013). Therefore, it is theorized that Asian American women are perceived as more typical of the Asian American group than Asian American men (Johnson et al., 2012). However, it is unclear how gender interacts with different Asian ethnicities. It is possible that because East Asian individuals are considered typical of the Asian American group, East Asian women are considered typical of the group. Therefore, participants may have been more likely to envision the Chinese applicant to be a woman than the Indian and Filipino applicants. We included a question for exploratory purposes in which we asked participants about their assumptions about the gender identity of the Asian applicants. A chi-square analysis showed that in both studies, participants were significantly more likely to think that the Chinese applicant identified as a “woman” instead of as a “man.” However, there was no significant difference in the perceived gender of the Indian and Filipino applicants^8^. Therefore, the perceived gender of the applicants may have played a role in determining scholarship worthiness, and future research should more directly test this possibility to understand how ethnic and gender typicality interact in participants’ perceptions of Asian Americans.

Finally, the studies were limited by their focus on a specific minority resource: scholarships. It is worth exploring whether perceived typical group members are generally more likely to be allocated minority resources than atypical group members. For example, are business loans more likely to get approved for small business owners of Chinese descent than owners of Indian and Filipino descent, if they are applying to use the loan to start an Asian restaurant? Future research may examine minority resource distributions beyond diversity awards to determine the generalizability of our findings to other domains.

## 5. Conclusions

It is important to consider how an institution’s lack of acknowledgement of the diversity within racial categories may influence social perceptions of that category. Indeed, structural and social influences may relate to observations that people in the U.S. tend to ignore the ethnic diversity of the Asian American category and instead default to thinking about East Asian Americans while overlooking South and Southeast Asian Americans (e.g., Vinluan & Remedios, 2024). We demonstrate the consequences of such typicality biases in the context of diversity scholarships, wherein participants used the perceived group typicality and group identity of Asian applicants to guide who they considered worthy of an Asian American scholarship. Furthermore, our results suggest that not everyone will be equally likely to receive the same academic opportunities. Decision-making processes for scholarship allocations warrant closer investigation to ensure that all members are given equal access to academic opportunities.

## Figures and Tables

**Table 1:** Descriptive statistics and correlations for Study 1 measures. * *p* < .05.

Measure	*M (SD)*	1.	2.	3.	4.
1. Competence	5.46 (0.91)				
2. Asian Identity	5.10 (1.49)	.323 *			
3. Asian Typicality	5.04 (1.51)	.196 *	.499 *		
4. Asian Scholarship Worthiness	5.03 (1.33)	.589 *	.458 *	.334 *	

**Table 2:** Descriptive statistics and correlations for Study 2 measures. * *p* < .05.

Measure	*M (SD)*	1.	2.	3.	4.
1. Competence	5.39 (0.90)				
2. Asian Identity	4.91 (1.57)	.248 *			
3. Asian Typicality	4.90 (1.55)	.113 *	.491 *		
4. Scholarship Worthiness	4.80 (1.33)	.549 *	.353 *	.179 *	

## Data Availability

Materials for both studies, including data and analysis syntax, are available on the Open Science Framework: https://osf.io/k4xng/overview?view_only=b8a4adcf307c45bea581166b49d802d3.

## Notes

1 At the time of data collection, this was the standard compensation rate.

2 We pre-registered that participants had to pass two attention checks; however, for one-third of our participants there was not a second attention check due to a programming error by the experimenter. Therefore, we only analyzed data from participants who passed one attention check.

3 We additionally asked participants to rate the applicants on perceived warmth (Fiske et al., 2002) and a series of Asian-specific stereotypes (e.g., foreign, good at STEM)—see Supplementary Materials for results.

4 All analyses are collapsed across the clustering variable.

5 At the time of data collection, this compensation rate was standard.

6 Note that the use of Bonferroni tests was not pre-registered in our analysis plan, but seemed to be the most reasonable path forward.

7 We decided to choose Cohen’s *d* = ±0.152 as our equivalence bound because *d* = 0.152 is the effect size for the Chinese–Indian comparison in the *Asian undergraduate* scholarship condition—the only significant comparison in this set of analyses.

8 In Study 1, the chi-square results for each applicant race condition are as follows: Chinese applicant condition, *X*^2^(1, *N* = 513) = 6.33, *p* = .012; Indian applicant condition, *X*^2^(1, *N* = 511) = .05, *p* = .825; and Filipino applicant condition, *X*^2^(1, *N* = 516) = 0.31, *p* = .860. In Study 2, the chi-square results for each applicant race condition are as follows: Chinese applicant condition, *X*^2^(1, *N* = 545) = 13.28, *p* < 0.001; Indian applicant condition, *X*^2^(1, *N* = 546) = .36, *p* = .549; and Filipino applicant condition, *X*^2^(1, *N* = 537) = .42, *p* = .517.

## Supplementary Materials

The following supporting information can be downloaded at: https://www.mdpi.com/article/10.3390/bs16060981/s1, File S1: Pilot Study: Essay Manipulation. ETS (2021) cited in the supplementary materials.
