# Supplementary material for: The Distribution of Asian American Scholarship Awards Among Chinese, Indian, and Filipino Individuals
Source: Behav Sci (Basel). 2026 Jun 12;16(6):981. doi: 10.3390/bs16060981 (PMC13295326; doi:10.3390/bs16060981)
Supplement: Supplementary file 1 [file behavsci-16-00981-s001.zip › behavsci-4220022-supplementary.pdf]

Online Supplement

Pilot Study: Essay Manipulation

For Studies 1 and 2, applicant ethnicity was manipulated by changing the identity participants read in each essay. We created three different personal statement essays for this study so that participants would not read the same essay written by the Chinese, Indian, and Filipino applicants, but so that each essay would be paired with each applicant's ethnicity. To ensure that participants saw each essay and applicant race only once, there were three clustering conditions. More specifically, in cluster 1, participants read essay 1 written by a Chinese writer, essay 2 written by an Indian writer, and essay 3 written by a Filipino writer. In cluster 2, participants read essay 1 by a Filipino writer, essay 2 by a Chinese writer, and essay 3 by an Indian writer. In cluster 3, participants read essay 1 by an Indian writer, essay 2 by a Filipino writer, and essay 3 by a Chinese writer. We piloted the essays so that the actual quality of the essays (i.e., how well the essay was written) did not differ when paired with either the Chinese, Indian, or Filipino identity. Furthermore, we wanted to make sure that the essays did not differ on three aspects of the scholarship we specified in Studies 1 and 2: scholarship, leadership, and community service.

Method

Participants and Design

The study was a 3 (*cluster*: 1, 2, 3) x 3 (*applicant ethnicity:* Chinese, Indian, Filipino) x 3 *essay*: 1, 2, 3) mixed-model design with applicant ethnicity and essay as repeated-measures factors. We decided to target a small-sized effect (d = 0.20) for 3 (*cluster*: 1, 2, 3) x 3 (*race:* Chinese, Indian, Filipino) interaction. A power analysis using PANGEA (Westfall, 2015) suggested we recruit at least n = 164 participants per between-subjects condition or a total of N = 492 participants. We recruited N = 558 participants from Amazon's Mechanical Turk in

exchange for $0.33 USD (63.8% White, 13.1% Black, 7.5% Asian, 5.6% Latinx; 41.9% male, 56.7% female; M_{age} = 33.8, SD_{age} = 11.72).

Procedure

Participants were told that they were going to be randomly assigned an essay prompt and that they would read and rate a series of essay excerpts. In reality, participants were assigned to the same essay prompt: "Some students have a background, identity, interest, or talent that perhaps hasn't always been important but, over time, has become so meaningful they believe their application would be incomplete without discussing it. If this sounds like you, then please share your story."

Participants were then randomly assigned to one of three *cluster* conditions. Within each *cluster* condition, participants were presented with three essays, one for each applicant ethnicity. For each essay, participants were asked to assess the essay quality with the following five items on a 7-point scale (*1* = strongly disagree; *7* = strongly agree): "Overall, the quality of the essay was excellent", "The essay was clear and concise", "The essay thoroughly answered the prompt", "The essay was well organized" and "The essay was written with minor grammatical errors." We based these items on the scoring guide for the analytical writing portion of the Graduate Record Examination (ETS, 2021). The previous five items were averaged into a composite score. Cronbach's alphas are listed in Table 1.

Table S1

Reliability for composite essay: overall quality score

Cluster Condition	Applicant Ethnicity		
	Chinese	Indian	Filipino
1	.83	.80	.84
2	.81	.80	.79
3	.74	.80	.74

We additionally assessed how well "The essay content demonstrated scholarship/leadership/community service" on a 7-point scale (*1* = strongly disagree; *7* = strongly agree). These items were mentioned in the description of the scholarship in the main manuscript. We then asked participants to rate "How masculine or feminine do you think the voice of the writer is?" (*1* = very masculine; *7* = very feminine) and "What do you think is the writer's high school GPA (out of 4.0)?" (0.0 – 4.0 in 0.5 increments). Finally, we told participants, "Imagine that this essay was written for an undergraduate college scholarship. To what extent do you agree or disagree with the following statement: The writer should be awarded a scholarship". Participants responded on a 7-point scale (*1* = strongly disagree; *7* = strongly agree).

Results

Data Analyses Plan

We conducted a 3 (*cluster*: 1, 2, 3) x 3 (*applicant ethnicity:* Chinese, Indian, Filipino) mixed-model Analysis of Variance on the items. The means and standard deviations for all the items are in Table 2.

Essay Quality

There was not a significant main effect of cluster on essay quality, $F(2,1096) = 0.58$, $p = .556$, $\eta^2_p = .001$, nor a significant main effect of race, $F(2,549) = 0.02$, $p = .979$, $\eta^2_p < .001$. There was a significant main interaction, $F(4,1096) = 3.73$, $p = .0051$, $\eta^2_p = .013$. However, Bonferroni-adjusted post hoc comparisons showed that there were not any significant comparisons.

Table S2

Pilot study results: descriptive statistics

Item	Cluster Condition	Applicant Ethnicity		
		Chinese *M(SD*	Indian *M(SD)*	Filipino *M(SD)*
Essay: Overall Quality	1	5.28 (1.03)	5.14 (1.02)	5.23 (1.06)
	2	5.13 (1.07)	5.29 (1.01)	5.26 (1.01)
	3	5.23 (0.93)	5.29 (1.02)	5.10 (1.01
	overall	5.21 (1.01)	5.24 (1.02)	5.19 (1.02)
Essay: Scholarship	1	4.86 (1.48)	4.89 (1.42)	4.70 (1.50)
	2	4.81 (1.52)	4.67 (1.56)	4.92 (1.46)
	3	4.76 (1.54)	4.89 (1.56)	4.85 (1.47)
	overall	4.81 (1.51)	4.82 (1.51)	4.82 (1.48)
Essay: Leadership	1	4.83 (1.41)	4.91 (1.44)	4.70 (1.52)
	2	4.96 (1.43)	4.61 (1.39)	4.85 (1.48)
	3	4.47 (1.57)	4.57 (1.63)	4.91 (1.48)
	overall	4.75 (1.48)	4.69 (1.50)	4.82 (1.49)
Essay: Community Service	1	5.57 (1.36)	5.47 (1.32)	5.37 (1.48)
	2	5.45 (1.40)	5.43 (1.56)	5.43 (1.53)
	3	5.31 (1.50)	5.40 (1.38)	5.38 (1.42)
	overall	5.44 (1.43)	5.43 (1.42)	5.39 (1.48)
Writer: Perceived Voice	1	4.57 (1.43)	4.20 (1.57)	4.18 (1.51)
	2	4.41 (4.24)	4.19 (1.48)	4.58 (1.38)
	3	4.24 (1.50)	4.28 (1.50)	4.25 (1.41)
	overall	4.41 (1.47)	4.22 (1.52)	4.33 (1.44)
Writer: Expected GPA	1	3.14 (0.59)	3.12 (0.56)	3.06 (0.58)
	2	3.03 (0.63)	3.04 (0.60)	2.96 (0.69)
	3	3.09 (0.64)	3.07 (0.63)	3.05 (0.61)
	overall	3.09 (0.62)	3.08 (0.60)	3.02 (0.63)
Overall Scholarship Worthiness	1	4.99 (1.28)	4.80 (1.47)	4.88 (1.31)
	2	4.92 (1.45)	4.99 (1.34)	5.02 (1.28)
	3	4.95 (1.39)	4.96 (1.41)	4.90 (1.39)
	overall	4.95 (137)	4.92 (1.41)	4.93 (1.33)

Scholarship Components

There was not a significant main effect of cluster on perceptions of scholarship, $F(2,546) = 0.05$, $p = .956$, $\eta^2_p < .001$, a significant main effect of race, $F(2,1092) = 0.07$, $p = .936$, $\eta^2_p < .001$, nor a significant interaction, $F(4,1092) = 2.35$, $p = .052$, $\eta^2_p = .009$.

There was not a significant main effect of cluster on perceptions of leadership, $F(2,544) = 1.36$, $p = .26$, $\eta^2_p = .005$, nor a significant main effect of race, $F(2,1088) = 1.37$, $p = .251$, $\eta^2_p = .003$. There was a significant interaction, $F(4,1088) = 7.26$, $p < .0001$, $\eta^2_p = .026$. Bonferroni-adjusted post hoc comparisons revealed that within cluster 3, participants rated the Filipino applicant's essay as demonstrating more leadership than the Chinese applicant's essay, $t(1086) = 3.95$, $p = .003$.

There was not a significant main effect of cluster on perceptions of community service, $F(2,540) = 0.44$, $p = .645$, $\eta^2_p = .002$, a significant main effect of race, $F(2,1080) = 0.08$, $p = .923$, $\eta^2_p < .001$, nor a significant interaction, $F(4,1080) = 1.18$, $p = .314$, $\eta^2_p = .004$.

Voice of Writer

There was not a significant main effect of cluster on perceived voice of the writer, $F(2,533) = 0.94$, $p = .391$, $\eta^2_p = .001$, nor a significant main effect of race, $F(2,1066) = 2.22$, $p = .109$, $\eta^2_p = .004$. There was a significant interaction, $F(4,1066) = 2.41$, $p = .048$, $\eta^2_p = .009$. However, Bonferroni-adjusted post hoc comparisons showed that there were not any significant comparisons.

GPA

There was not a significant main effect of perceived GPA of the writer, $F(2,537) = 1.93$, $p = .147$, $\eta^2_p = .007$. There was a significant main effect of race, $F(2,1074) = 3.51$, $p = .030$, $\eta^2_p = .006$. Participants rated the Chinese applicant as having a higher GPA ($M = 3.09$, $SD = 0.62$)

than the Filipino application (*M* = 3.02, *SD* = 0.63), *t*(1074) = 2.43, *p* = .045. There was not a significant interaction, *F*(4,1074) = 0.13, *p* = .973, $\eta^2_p < .001$.

Scholarship Worthiness

There was not a significant main effect of cluster, *F*(2,547) = 0.29, *p* = .751, $\eta^2_p = .001$, a significant main effect of race, *F*(2,1094) = 0.15, *p* = .858, $\eta^2_p < .001$, nor a significant interaction, *F*(4,1094) = 1.07, *p* = .370, $\eta^2_p = .004$.

Discussion

Overall, we concluded that participants were perceiving the essays as being equal in quality even when each the same essay was ostensibly written by a writer of a different racial identity. Although, there were a few significant main effects and interactions, there were not any consistencies in how the essays significantly differed from each other.

Study 1: Additional Measures

Method

In addition to the measures listed in the main manuscript, we asked participants to complete a series of stereotyping items. The warmth-related stereotypes were *friendly, well-intentioned, trustworthy, warm, good-natured,* and *sincere* (Fiske et al., 2002), which were averaged to make a composite warmth score ($\alpha_{Chinese}$ = .90, α_{Indian} = .89, $\alpha_{Filipino}$ = .90). The Asian American stereotypes were *hardworking, good at STEM-related fields, bilingual, foreign, traditional, family-oriented, shy,* and *reserved.* We decided to analyze the Asian American stereotypes as separate items because the Cronbach's alpha for a composite Asian American stereotype score was less than .80. All items were measured on a 7-point Likert scale (1 = *Strongly Disagree,* 7 = *Strongly Agree*). See Table S3 for descriptive statistics.

Results

We ran a series of separate one-way repeated measures ANOVA to examine if stereotype rating significantly differed by applicant ethnicity. See Table S3 for complete one-way ANOVA results. We followed up the significant main effects of applicant ethnicity with Bonferroni-adjusted post hoc comparisons and report them below.

There was a significant main effect of applicant ethnicity on the *good at STEM* stereotype, $F(2,1030) = 6.50$, $p = .002$, $\eta_p^2 = .012$. The Chinese applicant was rated as significantly better at STEM than the Filipino applicant, $F(1,515) = 12.25$, $p =.0005$, $\eta_p^2 = .023$, but not significantly better than the Indian applicant, $F(1,515) = 0.89$, $p = .347$, $\eta_p^2 = .002$. The Indian applicant was also rated as significantly better at STEM than the Filipino applicant, p = .040, 95% CI [0.004, 0.26].

Additionally, there was a significant main effect of applicant ethnicity on the *bilingual* stereotype, $F(2,1018) = 7.56$, $p = .0006$, $\eta_p^2 = .015$. The Chinese applicant was rated as more bilingual than both the Indian, $F(1,509) = 13.29$, $p = .0003$, $\eta_p^2 = .025$, and Filipino applicants, $F(1,509) = 6.26$, $p = .013$, $\eta_p^2 = .012$. There was not a significant difference in ratings between the Indian and Filipino applicants, $p = .504$, 95% CI [-0.21, 0.06].

Table S3

Study 1: descriptive statistics and one-way repeated-measures ANOVA results for stereotyping items

Stereotype	Applicant Ethnicity			One-way Repeated-Measures ANOVA Results
	Chinese *Mean (SD)*	Indian *Mean (SD)*	Filipino *Mean (SD)*	
Warmth	5.63 (0.89)	5.59 (0.91)	5.61 (0.91)	$F(2,1030) = 1.04$, $p = .354$, $\eta_p^2 = .002$
Hardworking	5.53 (1.11)	5.48 (1.15)	5.44 (1.19)	$F(2,1020) = 1.28$, $p = .279$, $\eta_p^2 = .003$
Good at STEM	4.64 (1.28)	4.60 (1.32)	4.47 (1.23)	$F(2,1030) = 6.50$, $p = .002$, $\eta_p^2 = .012$
Bilingual	5.24 (1.30)	5.02 (1.35)	5.09 (1.28)	$F(2,1018) = 7.56$, $p = .0006$, $\eta_p^2 = .015$
Foreign	4.92 (1.65)	4.88 (1.63)	4.96 (1.65)	$F(2,1016) = 1.05$, $p = .352$, $\eta_p^2 = .002$
Traditional	5.00 (1.35)	4.99 (1.37)	4.99 (1.36)	$F(2,1014) = 0.02$, $p = .983$, $\eta_p^2 < .0001$
Family-Oriented	5.69 (1.20)	5.66 (1.15)	5.65 (1.26)	$F(2,1026) = 0.24$, $p = .788$, $\eta_p^2 = .0004$
Shy	4.23 (1.53)	4.17 (1.52)	4.09 (1.58)	$F(2,1018) = 2.19$, $p = .113$, $\eta_p^2 = .004$
Reserved	4.59 (1.48)	4.59 (1.47)	4.52 (1.47)	$F(2,1016) = 0.75$, $p = .471$, $\eta_p^2 = .001$

Study 2: Additional Measures and Exploratory Analyses

Method

Participants in Study 2 were asked to complete the same measures as in Study 1. See Table S4 for descriptive statistics.

Results

Mixed-Model ANOVA

We ran a 2 (scholarship type) x 3 (applicant ethnicity) mixed-model ANOVA with applicant ethnicity as a repeated-measures factor. See Table S4 for complete ANOVA results. We followed up any significant main effects or interactions with Bonferroni-adjusted post hoc comparisons and report them below.

As in Study 1, there was a significant main effect of applicant race on the *good at STEM* stereotype, $F(2,1116) = 5.65$, $p = .004$, $\eta_p^2 = .010$. We replicated findings that the Chinese applicant ($M = 4.45$, $SD = 1.15$) was rated as significantly better at STEM than the Filipino applicant ($M = 4.30$, $SD = 1.13$), $F(1,558) = 9.97$, $p = .002$, $\eta_p^2 = .018$, but not the Indian applicant ($M = 4.44$, $SD = 1.17$), $F(1,558) = 0.12$, $p = .725$, $\eta_p^2 = .0002$. Additionally, the Indian applicant was rated as significantly better at STEM than the Filipino applicant, $p = .030$, 95% CI [0.01, 0.27].

Additionally, there was a significant main effect of applicant race on the *bilingual* stereotype, $F(2,1108) = 4.89$, $p = .008$, $\eta_p^2 = .009$. Participants rated the Chinese applicant ($M = 5.00$, $SD = 1.29$) as significantly more bilingual than both the Indian ($M = 4.83$, $SD = 1.33$), $F(1,554) = 9.12$, $p = .003$, $\eta_p^2 = .016$, and Filipino applicants ($M = 4.86$, $SD = 1.40$), $F(1,554) = 5.49$, $p = .019$, $\eta_p^2 = .010$. There was not a significant difference in ratings between the Indian and Filipino applicants, $p = 1.00$, 95% CI [-0.17, 0.11].

Table S4

Study 2: descriptive statistics and 2 (scholarship type: Asian, general) x 3 (applicant ethnicity: Chinese, Indian, Filipino) mixed-model ANOVA for all measures

Measure	Scholarship Type (ST)	Applicant Ethnicity (AE)			2x3 Mixed-Model ANOVA	
		Chinese *M(SD)*	Indian *M(SD)*	Filipino *M(SD)*		
Intelligence	Asian	5.40 (0.94)	5.39 (0.87)	5.36 (0.90)	ST	$F(1,559) = 0.07, p = .788, \eta_p^2 = .0001$
	General	5.39 (0.89)	5.43 (0.90)	5.38 (0.90)	AE	$F(2,1118) = 0.72, p = .487, \eta_p^2 = .001$
					STxAE	$F(2,1118) = 0.31, p = .734, \eta_p^2 = .001$
Asian Identity	Asian	5.31 (1.35)	4.51 (1.76)	5.11 (1.38)	ST	$F(1,540) = 1.88, p = .171, \eta_p^2 = .003$
	General	5.11 (1.42)	4.41 (1.77)	5.02 (1.47)	AE	$F(2,1080) = 51.09, p < .0001, \eta_p^2 = .086$
					STxAE	$F(2,1080) = 0.29, p = .749, \eta_p^2 = .001$
Asian Typicality	Asian	5.42 (1.25)	4.53 (1.73)	4.76 (1.41)	ST	$F(1,555) = 0.01, p = .913, \eta_p^2 < .0001$
	General	5.47 (1.31)	4.32 (1.76)	4.88 (1.38)	AE	$F(2,1110) = 95.89, p < .0001, \eta_p^2 = .147$
					STxAE	$F(2,1110) = 2.79, p = .062, \eta_p^2 = .005$
Warmth	Asian	5.51 (0.92)	5.51 (0.91)	5.59 (0.88)	ST	$F(1,559) = 0.52, p = .472, \eta_p^2 = .001$
	General	5.57 (0.87)	5.57 (0.89)	5.62 (0.88)	AE	$F(2,1118) = 2.58, p = .076, \eta_p^2 = .005$
					STxAE	$F(2,1118) = 0.11, p = .893, \eta_p^2 = .0002$
Hardworking	Asian	5.38 (1.09)	5.35 (1.13)	5.32 (1.11)	ST	$F(1,549) = 0.42, p = .516, \eta_p^2 = .001$
	General	5.38 (1.17)	5.38 (1.17)	5.44 (1.18)	AE	$F(2,1098) = 0.03, p = .971, \eta_p^2 < .0001$
					STxAE	$F(2,1098) = 0.84, p = .431, \eta_p^2 = .002$
Good at STEM	Asian	4.45 (1.20)	4.41 (1.19)	4.24 (1.20)	ST	$F(1,558) = 0.51, p = .476, \eta_p^2 = .001$
	General	4.45 (1.15)	4.46 (1.16)	4.36 (1.07)	AE	$F(2,1116) = 5.65, p = .004, \eta_p^2 = .010$
					STxAE	$F(2,1116) = 0.75, p = .473, \eta_p^2 = .001$

Table S4 (cont.)

Measure	Scholarship Type (ST)	Applicant Ethnicity (AE)			2x3 Mixed-Model ANOVA	
		Chinese *M(SD)*	Indian *M(SD)*	Filipino *M(SD)*		
Bilingual	Asian	4.95 (1.30)	4.84 (1.31)	4.84 (1.45)	ST	$F(1,554) = 0.14$, $p = .707$, $\eta_p^2 = .0003$
	General	5.05 (1.29)	4.82 (1.36)	4.88 (1.35)	AE	$F(2,1108) = 4.89$, $p = .008$, $\eta_p^2 = .009$
					STxAE	$F(2,1108) = 0.53$, $p = .588$, $\eta_p^2 = .001$
Foreign	Asian	4.91 (1.56)	4.75 (1.62)	4.80 (1.62)	ST	$F(1,556) = 0.58$, $p = .446$, $\eta_p^2 = .001$
	General	4.75 (1.71)	4.73 (1.70)	4.75 (1.67)	AE	$F(2,1112) = 1.50$, $p = .225$, $\eta_p^2 = .003$
					STxAE	$F(2,1112) = 0.75$, $p = .473$, $\eta_p^2 = .001$
Traditional	Asian	4.81 (1.38)	4.77 (1.42)	4.81 (1.39)	ST	$F(1,555) = 0.02$, $p = .894$, $\eta_p^2 < .0001$
	General	4.79 (1.41)	4.74 (1.40)	4.82 (1.35)	AE	$F(2,1110) = 0.45$, $p = .636$, $\eta_p^2 = .001$
					STxAE	$F(2,1110) = 0.06$, $p = .943$, $\eta_p^2 = .0001$
Family-Oriented	Asian	5.54 (1.16)	5.47 (1.27)	5.63 (1.16)	ST	$F(1,553) = 0.30$, $p = .582$, $\eta_p^2 = .001$
	General	5.58 (1.16)	5.53 (1.22)	5.65 (1.16)	AE	$F(2,1106) = 3.01$, $p = .050$, $\eta_p^2 = .005$
					STxAE	$F(2,1106) = 0.03$, $p = .972$, $\eta_p^2 < .0001$
Shy	Asian	4.12 (1.45)	3.92 (1.48)	3.89 (1.52)	ST	$F(1,553) = 0.02$, $p = .080$, $\eta_p^2 < .0001$
	General	3.98 (1.41)	3.97 (1.50)	3.93 (1.52)	AE	$F(2,1106) = 2.53$, $p = .880$, $\eta_p^2 = .005$
					STxAE	$F(2,1106) = 1.30$, $p = .272$, $\eta_p^2 = .002$
Reserved	Asian	4.33 (1.43)	4.27 (1.46)	4.10 (1.50)	ST	$F(1,547) = 0.72$, $p = .398$, $\eta_p^2 = .001$
	General	4.38 (1.38)	4.30 (1.39)	4.27 (1.42)	AE	$F(2,1094) = 3.67$, $p = .026$, $\eta_p^2 = .007$
					STxAE	$F(2,1094) = 0.63$, $p = .535$, $\eta_p^2 = .001$

Finally, there was a significant main effect of applicant race on the *reserved* stereotype, $F(2,1094) = 3.67$, $p = .026$, $\eta_p^2 = .007$. The Chinese applicant ($M = 4.36$, $SD = 1.40$) was rated as significantly more reserved than the Filipino applicant ($M = 4.19$, $SD = 1.46$), $F(1,547) = 7.17$, $p = .008$, $\eta_p^2 = .013$, but not the Indian applicant ($M = 4.28$, $SD = 1.42$), $F(1,547) = 1.37$, $p = .242$, $\eta_p^2 = .003$. There was not a significant difference in ratings between the Indian and Filipino applicants, $p = .379$, 95% CI [-0.06, 0.25].

Parallel Mediation Results

We conducted an exploratory parallel mediation analysis to test whether both perceived Asian identity and typicality mediated the relationship between applicant ethnicity and scholarship worthiness. We did not include perceived intelligence in the analysis because the ANOVA results indicated that there was no significant difference in perceived intelligence ratings by applicant ethnicity. Additionally, we collapsed across scholarship types because there was no significant interaction between applicant ethnicity and scholarship type on scholarship worthiness. Ethnicity (Chinese vs. Indian) significantly predicted Asian typicality ($\beta = -0.99$, 95% CI [-1.155, -0.90]) and Asian identity ($\beta = -0.74$, 95% CI [-0.90, -0.59]. However, unlike Study 1, only the indirect effect of ethnicity (Chinese vs. Indian) on scholarship worthiness via identity (*indirect effect* = - 0.32, 95% CI [-0.89, -0.20]) was significant, as the 95% confidence intervals did not include zero, while the indirect effect of ethnicity (Chinese vs. Indian) on scholarship worthiness via typicality (*indirect effect* = 0.25, 95% CI [-27.84, 29.45]) was not significant. These results suggest that only perceived Asian identity mediated the effect between ethnicity (Chinese vs. Indian) and scholarship worthiness. Ethnicity (Chinese vs. Filipino) significantly predicted only Asian typicality ($\beta = -0.60$, 95% CI [-0.73, -0.46]) but not Asian identity ($\beta = -0.12$, 95% CI [-0.28, 0.03]). Additionally, both the indirect effects of ethnicity

(Chinese vs. Filipino) on scholarship worthiness through typicality (*indirect effect* = 0.41, 95% CI [-44.12, 50.17) and identity (*indirect effect* = -0.06, 95% CI [-0.21, 0.01]) were not significant, as the 95% confidence intervals included zero. These findings suggest that neither Asian typicality nor identity mediated the effect between ethnicity (Chinese vs. Filipino) and scholarship worthiness.

We again conducted the same parallel mediation analyses, but this time separately by scholarship type. First, in the Asian undergraduate scholarship condition, ethnicity (Chinese vs. Indian) significantly predicted Asian typicality (β = -0.90, 95% CI [-1.10, -0.70]) and Asian identity (β = -0.76, 95% CI [-0.98, -0.54]. However, unlike Study 1, only the indirect effect of ethnicity (Chinese vs. Indian) on scholarship worthiness via identity (*indirect effect* = -0.37, 95% CI [-0.87, -0.22]) was significant, as the 95% confidence intervals did not include zero, while the indirect effect of ethnicity (Chinese vs. Indian) on scholarship worthiness via typicality (*indirect effect* = -0.04, 95% CI [-8.63, 13.75]) was not significant. These results suggest that only perceived Asian identity mediated the effect between ethnicity (Chinese vs. Indian) and scholarship worthiness. Ethnicity (Chinese vs. Filipino) significantly predicted only Asian typicality (β = -0.65, 95% CI [-0.85, -0.46]) but not Asian identity (β = -0.18, 95% CI [-0.39, 0.04]). Additionally, both the indirect effects of ethnicity (Chinese vs. Filipino) on scholarship worthiness through typicality (*indirect effect* = -0.03, 95% CI [-6.16, 9.59]) and identity (*indirect effect* = -0.09, 95% CI [-0.26, 0.02]) were not significant, as the 95% confidence intervals included zero. These findings suggest that neither Asian typicality nor identity mediated the effect between ethnicity (Chinese vs. Filipino) and scholarship worthiness.

Next, in the general undergraduate scholarship condition, ethnicity (Chinese vs. Indian) significantly predicted Asian typicality (β = -1.14, 95% CI [-1.37, -0.95]) and Asian identity (β =

-0.73, 95% CI [-0.94, -0.49]. However, unlike Study 1, only the indirect effect of ethnicity (Chinese vs. Indian) on scholarship worthiness via identity (*indirect effect* = -0.69, 95% CI [-1.25, -0.31]) was significant, as the 95% confidence intervals did not include zero, while the indirect effect of ethnicity (Chinese vs. Indian) on scholarship worthiness via typicality (*indirect effect* = 0.07, 95% CI [-0.17, 0.74]) was not significant. These results suggest that only perceived Asian identity mediated the effect between ethnicity (Chinese vs. Indian) and scholarship worthiness. Ethnicity (Chinese vs. Filipino) significantly predicted only Asian typicality (β = -0.58, 95% CI [-0.81, -0.37]) but not Asian identity (β = -0.10, 95% CI [-0.32, 0.16]). Additionally, both the indirect effects of ethnicity (Chinese vs. Filipino) on scholarship worthiness through typicality (*indirect effect* = 0.04, 95% CI [-0.09, 0.36]) and identity (*indirect effect* = -0.08, 95% CI [-0.38, 0.19]) were not significant, as the 95% confidence intervals included zero. These findings suggest that neither Asian typicality nor identity mediated the effect between ethnicity (Chinese vs. Filipino) and scholarship worthiness.

Figure S1

Parallel mediation results by A) scholarship type collapsed, B) Asian undergraduate scholarship condition only, and C) general undergraduate scholarship condition only. Bolded font indicates p<.05.

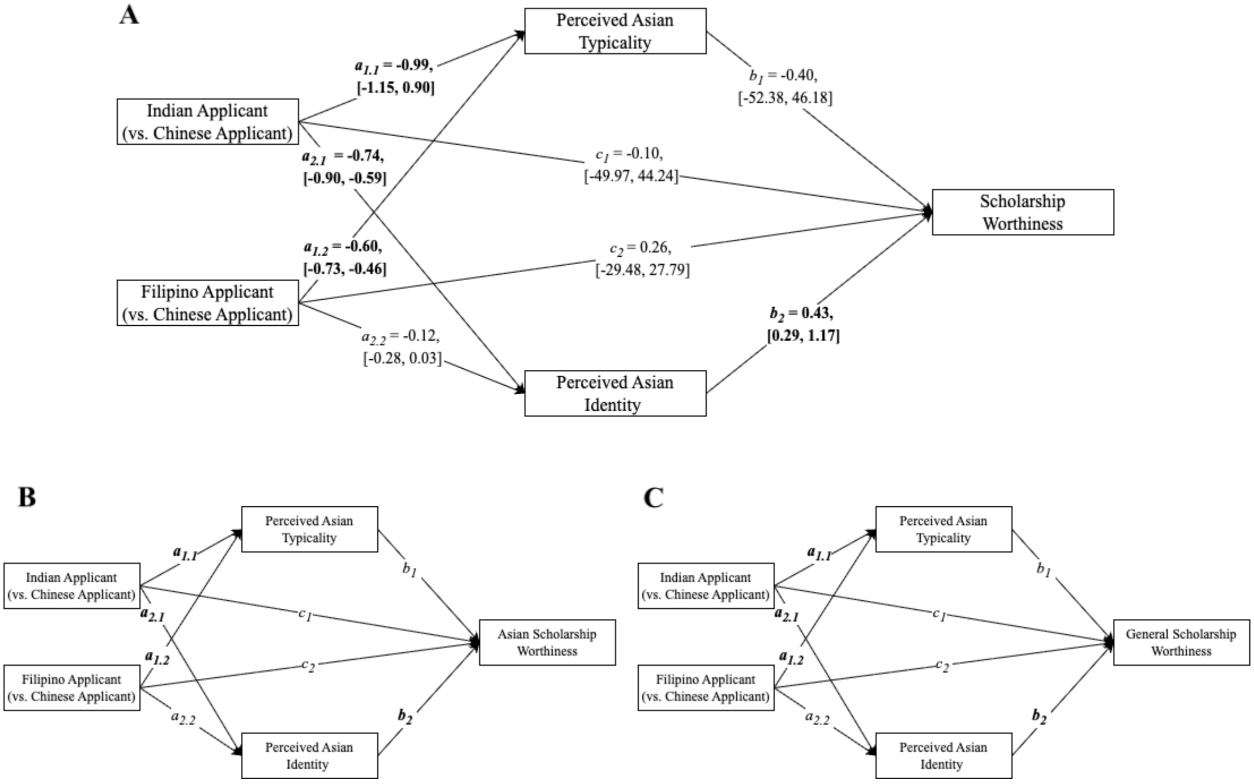

References

ETS (2021). *Scoring Guide for the Issue Task.* Retrieved from https://www.ets.org/gre/revised_general/prepare/analytical_writing/issue/scoring_guide

Westfall, J. (2015). PANGEA: Power analysis for general anova designs. Unpublished manuscript. *Available at http://jakewestfall.org/publications/pangea.pdf*
